# Triple Ectopic Gestation

**Published:** 1902-02

**Authors:** 


### Triple Ectopic Gestation.

Dr. Wilmer Krusen, in *American Medicine,* January 4th, reports a case of true unilateral triple tubal gestation, occurring in a woman 34 years old, who had delivered one child at term followed by five miscarriages in eight years. On June 12th, after an absence of menstruation for six or eight weeks, she was seized with an acute abdominal pain, associated with marked tenderness. This lasted two days. The symptoms now increased in severity, and a vaginal examination showed the uterus to be soft and enlarged, with a boggy sensation in the lateral and posterior vaginal formices. A diagnosis of ruptured ectopic gestation was made and an operation performed. A ruptured tubal sac was found, and three distinct, well formed fetuses removed. The writer believes this to be the only case of the kind ever reported. His mentions, however, are somewhat similarly reported by Sänger, of Leipsig, in 1893. R.

